# A New Malaria Agent in African Hominids

**DOI:** 10.1371/journal.ppat.1000446

**Published:** 2009-05-29

**Authors:** Benjamin Ollomo, Patrick Durand, Franck Prugnolle, Emmanuel Douzery, Céline Arnathau, Dieudonné Nkoghe, Eric Leroy, François Renaud

**Affiliations:** 1 Unité des Maladies Virales Émergentes, Centre International de Recherches Médicales de Franceville, Franceville, Gabon; 2 Laboratoire Génétique et Evolution des Maladies Infectieuses, UMR 2724 CNRS-IRD, IRD Montpellier, Montpellier, France; 3 Laboratoire de Paléontologie, Phylogénie & Paléobiologie, Institut des Sciences de l'Evolution (UMR 5554 CNRS), Université Montpellier II, Place E. Bataillon, Montpellier, France; 4 Unité Emergence des Pathologies Virales, UMR 190 IRD-Université de la Méditerranée, CIRMF, Franceville, Gabon; The Pennsylvania State University, United States of America

## Abstract

*Plasmodium falciparum* is the major human malaria agent responsible for 200 to 300 million infections and one to three million deaths annually, mainly among African infants. The origin and evolution of this pathogen within the human lineage is still unresolved. A single species, *P. reichenowi*, which infects chimpanzees, is known to be a close sister lineage of *P. falciparum*. Here we report the discovery of a new *Plasmodium* species infecting Hominids. This new species has been isolated in two chimpanzees (*Pan troglodytes*) kept as pets by villagers in Gabon (Africa). Analysis of its complete mitochondrial genome (5529 nucleotides including Cyt b, Cox I and Cox III genes) reveals an older divergence of this lineage from the clade that includes *P. falciparum* and *P. reichenowi* (∼21±9 Myrs ago using Bayesian methods and considering that the divergence between *P. falciparum* and *P. reichenowi* occurred 4 to 7 million years ago as generally considered in the literature). This time frame would be congruent with the radiation of hominoids, suggesting that this *Plasmodium* lineage might have been present in early hominoids and that they may both have experienced a simultaneous diversification. Investigation of the nuclear genome of this new species will further the understanding of the genetic adaptations of *P. falciparum* to humans. The risk of transfer and emergence of this new species in humans must be now seriously considered given that it was found in two chimpanzees living in contact with humans and its close relatedness to the most virulent agent of malaria.

## Author Summary

In 2002, the publication of the genome of *Plasmodium falciparum*, the most malignant agent of malaria, generated hopes in the fight against this deadly disease by the opportunities it offered to discover new drug targets. Since then results have not lived up to the expectations. The development of comparative genomics to further understanding of *P. falciparum* has indeed been hindered by a lack of knowledge of closely related species' genomes. Only one species, *P. reichenowi*, infecting chimpanzees, was hitherto known as a sister lineage of *P. falciparum*. Here we describe a new *Plasmodium* species infecting chimpanzees in Africa. Based on its whole mitochondrial genome, we demonstrate that this species is a relative of *P. falciparum* and *P. reichenowi*. The analysis of its genome should thus offer the opportunity to explore *P. falciparum* specific adaptations to humans. Our results bring new elements to the debate surrounding the origin of this lineage. They suggest that it may have been present in early hominoids and may have experienced a radiation congruent with that of its hosts. Our discovery highlights the paucity of our knowledge on the richness of *Plasmodium* species infecting primates and calls for more research in this direction.

## Introduction

Malaria is a major parasitic worldwide scourge, infecting and killing several million people each year [1]. Among the numerous *Plasmodium* species that infect reptiles, birds and mammals, four of them are human-specific: *P. falciparum*, *P. vivax*, *P. malariae* and *P. ovale*. The most virulent agent is *P. falciparum*, which kills up to three million people each year, mainly in Africa [1]. In spite of persistent control efforts set up since the end of the fifties, the disease is far from being under control. Even though numerous articles are published every year about the parasite and the disease, progress in controlling malaria has been limited. Resistance has evolved against virtually all drugs currently available [2], so that the disease frequently reemerges in different parts of the world [3],[4].

The recent availability of complete *Plasmodium* genomes [5]–[9] has generated new hopes in the fight against this parasite. Thanks to their comparison we have now a far better understanding of their genomic architecture and of the genes that may help the parasite to escape the host immune response [5]–[10]. This approach remains unfortunately limited regarding the main malignant agent of malaria, *P. falciparum*. One problem is the lack of knowledge about other closely related apicomplexan models that can serve as reference and comparison [11]. At present, only one species, *P. reichenowi* is known as a close sister lineage of *P. falciparum* [8],[12],[13]. Other *Plasmodium* species (*P. rodhaini* and *P. schwetzi*) were in the past described as parasites of the African great apes (i.e. chimpanzee and gorilla), but they were considered as closely related, for the first, to *P. malariae* and, for the second, to *P. vivax* [14] or *P. ovale* [15], which are very divergent from *P. falciparum* [12],[13],[16]. The development of comparative genomics for *P. falciparum* depends therefore on obtaining additional information about the diversity of *P. reichenowi* [17] and other *Plasmodium* species parasitic to the African Great Apes-Human lineage (the AGAH-lineage), currently represented by only two known species, *P. falciparum* and *P. reichenowi*.

In this manuscript, we report the discovery of a new *Plasmodium* species infecting Hominids in Africa. This new species was isolated from two chimpanzees and is a close relative of *P. falciparum*, the most virulent agent of human malaria.

## Results/Discussion

To explore the diversity of species belonging to the *Plasmodium* AGAH-lineage in Africa, we collected blood samples from 17 chimpanzees recently trapped from the wild and kept as pets in villages of Gabon by hunters and their families (see Figure S1). Considering that only the subspecies *Pan troglodytes troglodytes* has been found in Gabon, these 17 animals are likely to belong to this chimpanzee subspecies.

Among them, two were found to be infected with *Plasmodium* by means of PCR assay or microscopy. The other 15 animals were found negative both by microscopy and PCR assay. For these two chimpanzees (named B and K), observed parasites under microscopy were *falciparum*-like (ring stages with two chromatin dots and presence of multiply-infected red blood cells [15]). Thick blood smears revealed low parasitemia in both individuals, approximately 300 parasites/µl for chimpanzee B and 2000 parasites/µl for chimpanzee K.

For both, we amplified and sequenced the parasite's *Cytochrome* b (*Cyt* b) gene. The *Cyt* b sequences obtained were similar between the two samples (identity of 99.8% based on 866 nucleotides (nt)), but different from all other *Plasmodium Cyt* b sequences known to date. The most similar sequences obtained using BLAST were *Cyt* b sequences from *P. reichenowi* and *P. falciparum*, which show 92% and 91% identity, respectively.

Because the *Cyt* b sequences were partial, we studied the whole mitochondrial DNA (mtDNA) of these two new isolates (named *P. sp_K* and *P. sp_B*). For isolate K, we amplified 5529 nt including three main genes: *Cytochrome oxydase* I (*Cox* I), *Cytochrome oxydase* III (*Cox* III) and *Cytochrome b* (*Cyt* b). Apart from short missing segments amounting to 420 nt, the mtDNA sequenced corresponds to the whole *P. falciparum* 3D7 mtDNA (5949 nt). For technical reasons (certainly due to the very low parasitemia and degraded DNA), we were unable to accomplish this sequencing for isolate B.

In order to determine the evolutionary relationships of this new *Plasmodium* relative to other species, we compared its sequence to 17 known complete *Plasmodium* mitochondrial genome sequences, with the bird apicomplexan parasite *Leucocytozoon caulleryi* as an outgroup (see Table S1). Maximum likelihood (ML) phylogenetic trees were reconstructed at both the nucleotide and amino acid levels on the whole mitochondrial genome sequence, considered as a single genetic unit [18].

**Figure 1:**
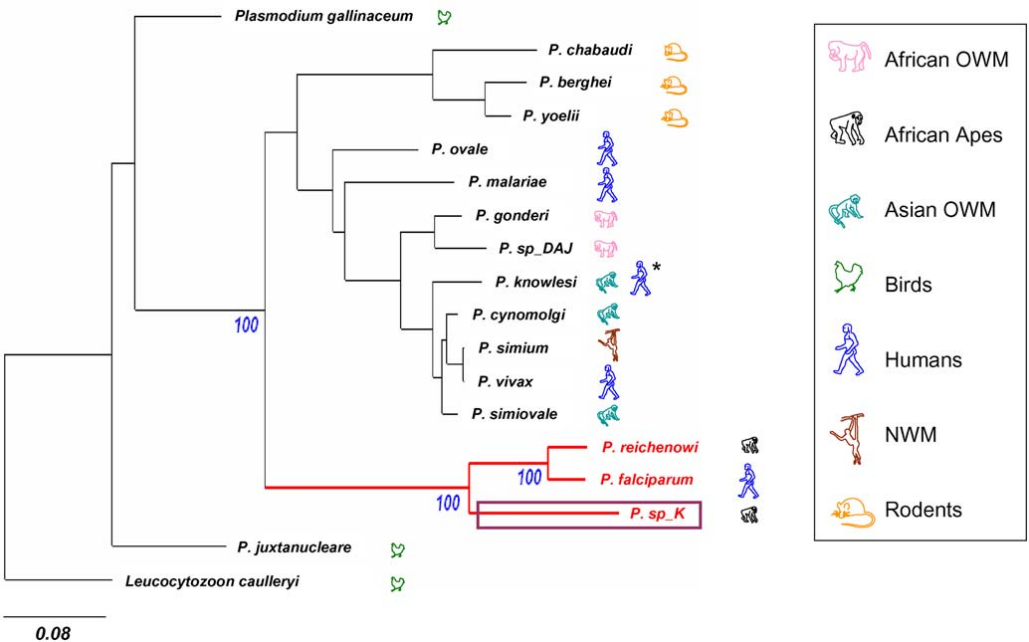
Phylogenetic relationships among *Plasmodium* species (including *P. sp_K*) and associated host groups. The phylogram presented here was reconstructed by a Maximum Likelihood approach from whole mitochondrial DNA sequence data. Bootstrap values obtained are only shown (in blue) for the nodes inside the African Great Apes/Human lineage (represented in red). *Leucocytozoon caulleryi* was used as outgroup. Scale bar shows 0.08 substitutions per site. OWM: Old World Monkeys; NWM: New World Monkeys. The purple box highlights the new species (*P. sp_K*) discovered in chimpanzees in Gabon. *P. knowlesi, which naturally infects macaques in Southeast Asia, is now considered as the “fifth human malaria agent” because of a recent shift to humans.

DNA and protein analyses provided identical results: the parasite collected in chimpanzee K belongs to the AGAH-lineage but is more divergent from *P. falciparum* than is *P. reichenowi* (Figure 1; see also Figure S3 for the tree reconstructed from the partial *Cyt* b sequence (866 nt) and including both *P. sp*_K and *P. sp*_B). Over the entire mitochondrial genome, the genetic distance observed between the new taxon (*P. sp_K*) and *P. falciparum* (*d* = 0.213 substitutions per nucleotide site on the ML phylogram) or *P. reichenowi* (*d* = 0.215) is almost four times higher than the distance observed between *P. falciparum* and *P. reichenowi* (*d* = 0.058).

To estimate the divergence time of the plasmodium AGAH-lineage, we used a calibration chosen within the hominid hosts. The age of the *P. falciparum*/*P. reichenowi* split is generally considered to be similar to the one separating humans from chimpanzees [12],[13],[19], that is, between four and seven million years [20]–[22]. Because of pervasive variations of mitochondrial substitution rates among malaria parasite lineages (Figure 1), a Bayesian relaxed molecular clock was used, which revealed a divergence time of 21±9 Myrs between the new *Plasmodium* species and the clade constituted by *P. falciparum* and *P. reichenowi*. Interestingly, this estimated time frame fits with the radiation of hominoids during the Miocene [23]. Our results suggest therefore that the plasmodium AGAH-lineage may have been present in early hominoids [23] and that this lineage may have also experienced a diversification during the early Miocene period as it occurred for their hosts [23]. Obviously, this estimated time of divergence is dependent on the calibration used. Recently, Martin and colleagues [24] suggested that the split between *P. falciparum*/*P. reichenowi* might have occurred far earlier than previously considered. They propose that *P. falciparum* originated from a recent transfer of *P. reichenowi* to humans during the last 2.8 Myrs [25]. Under this hypothesis, the new species would have diverged from *P. falciparum/P. reichenowi* about 10 Myrs ago. As divergence data are lacking for those parasites from the fossil record, we are unable to distinguish between these two hypotheses. Further data on the diversity of *Plasmodium* infecting great apes in Africa will certainly help resolve this particular aspect of the evolution of *P. falciparum*.

In conclusion, we bring to light the existence of a new *Plasmodium* species that infects chimpanzees in Gabon. We propose to name this new species *Plasmodium gaboni* sp. nov. in reference to the country where we obtained it. Our discovery suggests that great apes and perhaps simian primates may host a far higher diversity of *Plasmodium* species in Africa than previously recognised. Beyond the interest of this new species in the understanding of the evolution of this group of parasites, its position in the AGAH-lineage as the sister-group of *P. falciparum*/*P. reichenowi* opens up the possibility of exploring lineage-specific evolution using comparative genomics, and hence, to look for the genes responsible for the adaptation of these parasites to their specific hosts. Comparison between genomes will advance understanding of the differences in pathology and the processes at work in the interaction with the vertebrate or the mosquito hosts [9],[17]. It is thus essential to complete the nuclear genome sequence of this new species of phylogenetic importance within the AGAH-lineage, in order to enhance our knowledge of the functional genomics of human malaria parasites.

Finally, this new species was discovered in two chimpanzees conserved as pets by villagers in Gabon. Given the recent history of primate to human shifts in several pathogens (e.g. HIV [26]; Ebola [27]; for *Plasmodium*, the most recent involved *P. knowlesi* and occurred from macaques to humans in Asia [28]) and the close proximity between *P. gaboni* and the most virulent agent of malaria, *P. falciparum*, we think that the risk of transfer of this species to humans must be seriously considered.

## Materials and Methods

### Origin of parasite samples, microscopic analyses and DNA extraction

Blood aliquots of 17 chimpanzees were collected from different parts of Gabon (Figure S1). The samples were collected from wild-born animals kept as pets by hunters and their families. The investigation was approved by the Government of the Republic of Gabon and by the Animal Life Administration of Libreville, Gabon (no. CITES 00956). All animal work has been conducted according to relevant national and international guidelines. Blood samples were collected in 7 ml EDTA vacutainers from chimpanzees under ketamine anaesthesia. Clots and plasma were obtained by centrifugation and stored at −20°C until they were transported to the Centre International de Recherches Médicales de Franceville (CIRMF), Gabon, where they were stored at −80°C until processed for testing. Total DNA (*Plasmodium* and host) was isolated and purified using the DNeasy blood kit (Qiagen, Hilden, Germany) according to the manufacturer's instructions. The DNA was eluted in 100 µl of sterile water.

Microscopic analyses of the blood samples by thin blood smears revealed that two chimpanzees were infected by *falciparum*-like parasites (ring stages with two chromatin dots and presence of multiply-infected red blood cells [15]). The two infected individuals were respectively a young chimpanzee (*Pan troglodytes*, male of 3 years old) from the area of Bakoumba (1°28′0″S/13°0′0″E, Haut-Ogooué Province) and a young chimpanzee (*Pan troglodytes*, male of 2–3 years old) from the village of Koulamoutou (1°23′59″S/12°13′0″E, Ogooué-Lolo Province) (Figure S1). The two *Plasmodium* isolates were named *P. sp_B* and *P. sp_K*, respectively.

In both chimpanzees (named B and K), parasitemia were estimated using thick blood smears. The number of parasites per microliter was estimated using the ratio of the number of observed parasites by the number of observed lymphocytes. To extrapolate to the number of parasites per microliter, we considered that there were about 8000 lymphocytes per microliter of whole blood. The infection status of the two chimpanzees was then confirmed by PCR of the *Cytochrome* b gene (see below for PCR conditions).

### PCR conditions

To obtain whole mitochondrial genome sequences from *P. sp_B* and *P. sp_K*, seven primer pairs were designed based on the mitochondrial genome sequence of *Plasmodium falciparum* (GenBank accession number AY282930) using Primer 3 (v. 0.4.0) [29] and eight primer pairs already published [30] (Table S2). PCR amplification and sequencing were performed on seven and eight overlapping regions covering a complete linear copy of the mtDNA genome, respectively. The *Cytochrome oxydase* I (*Cox* I), the *Cytochrome oxydase* III (*Cox* III) and the *Cytochrome* b (*Cyt* b) genes were also amplified specifically using published primers [30]–[32].

All amplification reactions were performed using a MJ Research PTC100 thermal cycler.

- The amplifications with each of the eight primer pairs were performed using conditions previously published [30];
- The amplifications with each of the seven primer pairs for *Cox* I and *Cox* III genes were performed in a volume of 25 µl with the following components: 2.5 µl PCR reaction buffer 10× including 1.5 mM MgCl_2_, 240 µM of each dNTPs, 20 pmol of each primer, 1 U Taq DNA polymerase (Roche Diagnostics, Laval, Canada), and 2 µl solution of DNA template. Temperature cycling was as follows; 94°C for 2 min, 94°C for 20 sec, 52°C for 10 sec, 48°C for 10 sec, 60°C for 1 min 30 sec for 38 cycles.
- For the amplifications (nested PCR) of the first round of *Cyt* b gene, we used 2 µl of DNA template in a 20 µl reaction volume, containing : 4 µl of 5× Reaction Buffer, 1.5 mM MgCl_2_, 200 µM of each dNTP, 20 pmol of each primer (DW2 and DW4), and 2.5 U Taq DNA Polymerase (Promega). Cycling conditions for the first round were as follows : 3 min at 94°C; 20 sec at 94°C; 20 sec at 60°C; 1 min 30 sec at 72°C (repeated for 35 cycles); 10 min at 72°C. For the second round of *Cyt* b gene amplification we used 1 µl of 1^st^ PCR template in a 25 µl reaction volume, containing : 5 µl of 5× buffer, 1.25 mM MgCl_2_, 250 µM of each dNTP, 37.5 pmol of each primer (CYTb1 and CYTb2), and 0.5 U Taq DNA Polymerase (Invitrogen, San Diego, US). Cycling conditions for the second round were as follows : 5 min at 95°C; 30 sec at 94°C; 30 sec at 45°C; 1 min 30 sec at 72°C (repeated for 35 cycles); 10 min at 72°C.

The amplified products (5 µl) were run on 1.5% agarose gels in TAE buffer to detect the correct band. The PCR-amplified products were used as templates for sequencing. DNA sequencing was performed by CoGenics Genome Express (Meylan, France).

### Alignment and analysis of mitochondrial genomes

For *P. sp_B*, we were only able to amplify and sequence a part of *Cyt* b (866 nt) (deposited in the GenBankTM Database under the accession number FJ895308). For *P. sp_K*, sequences obtained from all primer datasets were aligned and compared using ClustalW (v 1.8.1 in BioEdit v.7.0.9.0. software [33]) and a mtDNA consensus sequence of *P. sp_K* was created (GenBank Accession number FJ895307; see Figure S2).

In comparative analyses, we used 17 previously published mitochondrial genome sequences from *P. falciparum*, *P. reichenowi*, *P. gallinaceum*, *P. juxtanucleare*, *P. knowlesi*, *P. simiovale*, *P. simium*, *P. vivax*, *P. cynomolgi*, *P. yoelii*, *P. berghei*, *P. chabaudi*, *P. ovale*, *P. malariae*, *P. gonderi*, and *P.sp*. DAJ-2004, with *Leucocytozoon caulleryi*, an avian malaria parasite used here as outgroup. Hosts and GenBank accession numbers for these taxa are given in the Table S1. The multiple alignment of the 18 sequences was conducted using ClustalW (see e.g. Figure S2).

Phylogenetic relationships between mtDNA haplotypes (for whole mitochondrial genome) were inferred from all codon positions and non-coding regions. Non-sequenced sites and sites with gaps (or missing sites) (when gaps were present in more than 5% of the species) were removed, yielding a total of 5 805 sites available for subsequent inferences.

Maximum Likelihood (ML) tree reconstruction was conducted from the whole mitochondrial genome. For this, *Cyt* b, *Cox* I, *Cox* III, and non-coding sequences were concatenated and analysed under a single model of nucleotide or amino acid evolution. The best-fitting ML model under the Akaike Information Criterion was GTR (General Time Reversible)+Γ (Gamma distribution)+I (Invariable sites's distribution) for nucleotides as identified by ModelTest [34] and mtART (replacement matrix developed for arthropod mitochondrial proteins)+Γ+I for amino acids as identified by ProtTest [35]. The highest-likelihood DNA and protein trees and corresponding bootstrap support values were obtained by PhyML (freely available at the ATGC bioinformatics platform http://www.atgc-montpellier.fr/) using NNI (Nearest Neighbor Interchange)+SPR (Subtree Pruning Regrafting) branch swapping and 100 bootstrap replicates [36].

### Estimation of divergence time

The ML analysis evidenced pervasive variations of mitochondrial DNA substitution rates among malaria parasite lineages. In this context, we used a Bayesian relaxed molecular clock approach to estimate the divergence times of *Plasmodium* species. The log-normal rate-autocorrelated model [37] was adopted to relax the molecular clock hypothesis as it has been shown to reasonably fit various data sets [38]. We assumed a calibration interval of 4–7 Myrs for the split between *P. falciparum* and *P. reichenowi* [12],[19] to reflect the one among their human and chimpanzee hosts [20]–[22]. Dating estimates were computed by the Bayesian procedure implemented in the PhyloBayes software [38],[39], version 3.0 (http://www.atgc-montpellier.fr/phylobayes/), with a uniform prior on root age and divergence times. We used the CAT Dirichlet process with the number of components, weights and profiles all inferred from the ML topology, with a general time reversible (GTR) matrix of exchangeability among nucleotides, and a 4-category discrete Gamma (Γ) distribution of substitution rates across sites. Two independent MCMC runs were conducted for 1,000,000 generations, with sampling every 10 cycles. After a burn-in of 100 cycles, divergence times were computed, and were virtually identical for the two chains.

## Supporting Information

Figure S1 Location of the 17 sampled chimpanzees (*Pan troglodytes*) in Gabon. Each circle represents a unique sample. The fifteen uninfected chimpanzees are shown in yellow and the two infected ones in red. These latter two were collected in the villages of Koulamoutou (Ogooué-Lolo province) and Bakoumba (Haut Ogooué province), respectively.
(4.88 MB TIF)

Figure S2 Multiple sequence alignment of the whole mitochondrial DNA of the three species *P. falciparum* (3D7 strain), *P. reichenowi* and *P. sp_K* using CLUSTAL W (v. 1.81). A dot indicates an identical nucleotide and a dash indicates a gap compared to the *P. falciparum* reference sequence. Degenerate nucleotides as follows: W = AT, Y = CT, K = GT, M = AC.
(7.36 MB TIF)

Figure S3 Phylogenetic relationships among *Plasmodium* species (including *P. sp_K* and *P. sp_B*). The phylogram presented here was reconstructed by a Maximum Likelihood approach from partial *Cyt b* DNA sequence data (866 nt). Bootstrap values obtained are only shown (in blue) for the nodes inside the African Great Apes - Human lineage (represented in red). *Leucocytozoon caulleryi* was used as outgroup. Scale bar shows 0.09 substitutions per site.
(1.87 MB TIF)

Table S1 Parasite species used in this study with GenBank accession numbers and a description of their natural hosts.
(0.04 MB DOC)

Table S2 Amplification primers of the mitochondrial genome. Primers used in this study for the amplification of the whole mitochondrial genome and the three genes (*Cox* I, III and *Cyt* b) separately for *P.sp_B* and *P.sp_K* based on the mitochondrial genome sequence of *P. falciparum* 3D7 strain (GenBank Acc. no. AY282930). The fragment sizes are also estimated from the *P. falciparum* 3D7 sequence. mt: mitochondrial; F: Forward; R: Reverse; bp: base pairs; *Cox* I and *Cox* III: cytochrome oxydase I and III, respectively; *Cyt* b: cytochrome b. *Cox* I and *Cyt* b are amplified by nested PCR.
(0.06 MB DOC)

Text S1 Supporting figures and tables.
(1.05 MB DOC)
