# Supplementary figures and images for: A New Malaria Agent in African Hominids
Source: PLoS Pathog. 2009 May 29;5(5):e1000446. doi: 10.1371/journal.ppat.1000446 (PMC2680981; doi:10.1371/journal.ppat.1000446)

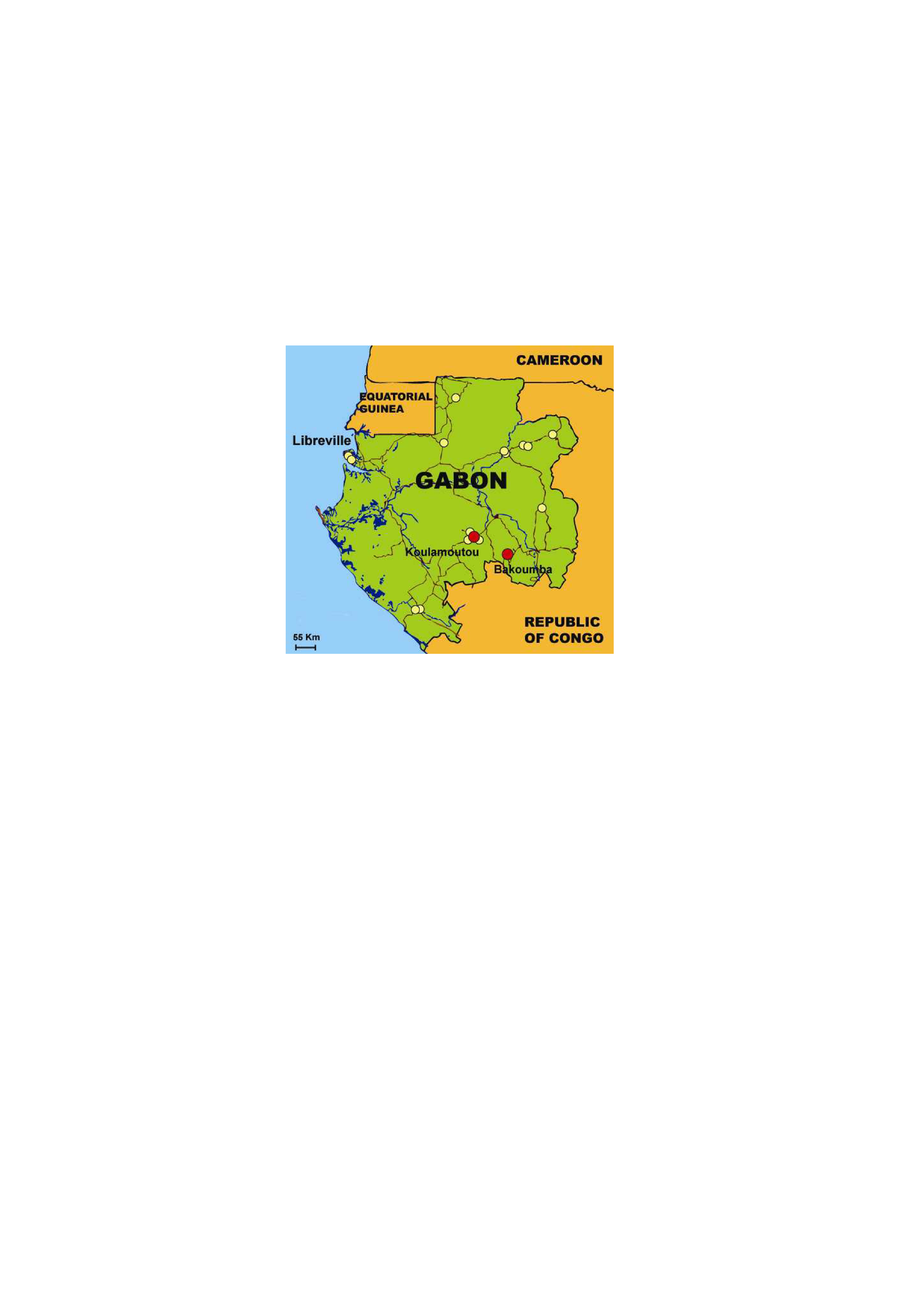

Supplement: Figure S1 — Location of the 17 sampled chimpanzees (Pan troglodytes) in Gabon. Each circle represents a unique sample. The fifteen uninfected chimpanzees are shown in yellow and the two infected ones in red. These latter two were collected in the villages of Koulamoutou (Ogooué-Lolo province) and Bakoumba (Haut Ogooué province), respectively. (4.88 MB TIF) [file ppat.1000446.s001.tif]

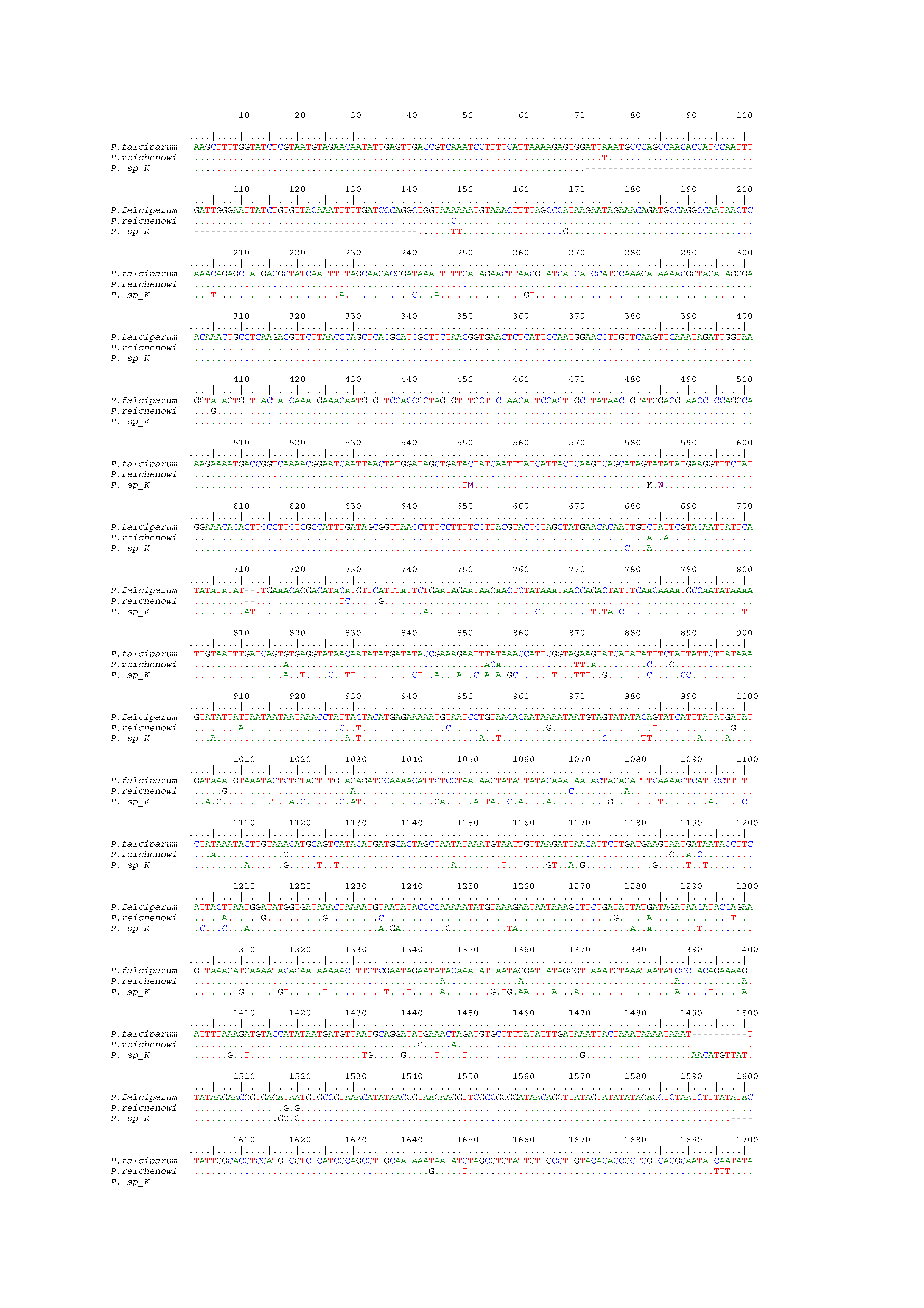

Supplement: Figure S2 — Multiple sequence alignment of the whole mitochondrial DNA of the three species P. falciparum (3D7 strain), P. reichenowi and P. sp_K using CLUSTAL W (v. 1.81). A dot indicates an identical nucleotide and a dash indicates a gap compared to the P. falciparum reference sequence. Degenerate nucleotides as follows: W = AT, Y = CT, K = GT, M = AC. (7.36 MB TIF) [file ppat.1000446.s002.tif]

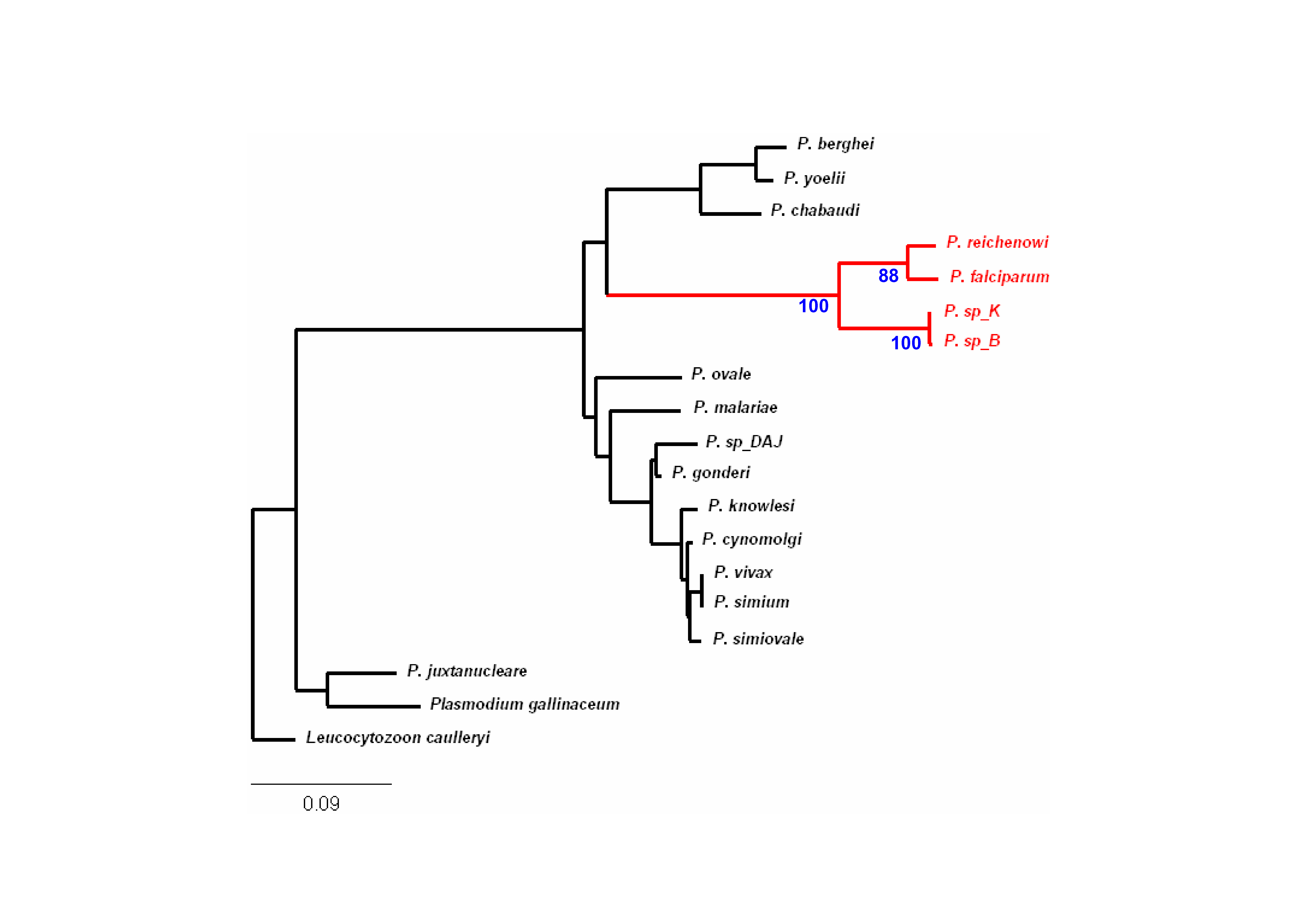

Supplement: Figure S3 — Phylogenetic relationships among Plasmodium species (including P. sp_K and P. sp_B). The phylogram presented here was reconstructed by a Maximum Likelihood approach from partial Cyt b DNA sequence data (866 nt). Bootstrap values obtained are only shown (in blue) for the nodes inside the African Great Apes - Human lineage (represented in red). Leucocytozoon caulleryi was used as outgroup. Scale bar shows 0.09 substitutions per site. (1.87 MB TIF) [file ppat.1000446.s003.tif]
