# Supplementary material for: A New Malaria Agent in African Hominids
Source: PLoS Pathog. 2009 May 29;5(5):e1000446. doi: 10.1371/journal.ppat.1000446 (PMC2680981; doi:10.1371/journal.ppat.1000446)
Supplement: Table S1 — Parasite species used in this study with GenBank accession numbers and a description of their natural hosts. (0.04 MB DOC) [file ppat.1000446.s004.doc]

**table S1. Parasite species used in this study with GenBank accession numbers and a description of their natural hosts.**

| **Species** | **Natural host** | **GenBank Accession numbers** |
| --- | --- | --- |
| *Plasmodium falciparum (3D7)* | Human | AY282930 |
| *P. vivax* | Human | NC_007243 |
| *P. ovale* | Human | AB354571 |
| *P. malariae* | Human | AB354570 |
| *P. reichenowi* | Chimpanzee and Gorilla | NC_002235 |
| *P. sp_K* | Chimpanzee | FJ895307 this study |
| *P. sp_B* | Chimpanzee | FJ895308 this study |
| *P. gonderi* | African Old World Monkey | AB434918 |
| *P. sp. DAJ-2004* | African Old World Monkey | AY800112 |
| *P. knowlesi* | Asian Old World Monkey and Human | AY722797 |
| *P. simium* | New World Monkey | NC_007233 |
| *P. cynomolgi* | Asian Old World Monkey | AY800108 |
| *P. simiovale* | Asian Old World Monkey | AB434920 |
| *P. chabaudi* | Rodent | AF014116 |
| *P. yoelii* | Rodent | M29000 |
| *P. berghei* | Rodent | AF014115 |
| *P. juxtanucleare* | Bird | AB250415 |
| *P. gallinaceum* | Bird | AB250690 |
| *Leucocytozoon caulleryi* | Bird | AB302215 |
