# Supplementary material for: A New Malaria Agent in African Hominids
Source: PLoS Pathog. 2009 May 29;5(5):e1000446. doi: 10.1371/journal.ppat.1000446 (PMC2680981; doi:10.1371/journal.ppat.1000446)
Supplement: Table S2 — Amplification primers of the mitochondrial genome. Primers used in this study for the amplification of the whole mitochondrial genome and the three genes (Cox I, III and Cyt b) separately for P.sp_B and P.sp_K based on the mitochondrial genome sequence of P. falciparum 3D7 strain (GenBank Acc. no. AY282930). The fragment sizes are also estimated from the P. falciparum 3D7 sequence. mt: mitochondrial; F: Forward; R: Reverse; bp: base pairs; Cox I and Cox III: cytochrome oxydase I and III, respectively; Cyt b: cytochrome b. Cox I and Cyt b are amplified by nested PCR. (0.06 MB DOC) [file ppat.1000446.s005.doc]

**table S2.** **Amplification primers of the mitochondrial genome**. Primers used in this study for the amplification of the whole mitochondrial genome and the three genes (*Cox* I, III and *Cyt* b) separately for *P.sp_B and P.sp_K* based on the mitochondrial genome sequence of *P. falciparum* 3D7 strain (GenBank Acc. no. AY282930)*.* The fragment sizes are also estimated from the *P. falciparum* 3D7 sequence. mt: mitochondrial; F: Forward; R: Reverse; bp: base pairs; *Cox* I and *Cox* III: cytochrome oxydase I and III, respectively; *Cyt* b: cytochrome b. *Cox* I and *Cyt* b are amplified by nested PCR.

| **Primer Name** | **Sense** | **Sequence (5''3')** | **Fragment size** |
| --- | --- | --- | --- |
| **Whole mt genome:** |  |  |  |
| Pfmito/F1 | F | CTCTCTCGATTTCCAGATGTTG | 821bp |
| Pfmito/R1 | R | GCTGGGCATTTAATCCACTC |  |
| Pfmito/F2 | F | ATTAAATGCCCAGCCAACAC | 888bp |
| Pfmito/R2 | R | TGTGTTACAGGATTACATTTTTCTCA |  |
| Pfmito/F3 | F | AATACTCTGTAGTTTGTAGAGATG | 885bp |
| Pfmito/R3 | R | CCTTTAATGTAGTTTCCTCA |  |
| Pfmito/F4 | F | TGGACCGAATAAAGCTGTGA | 885bp |
| Pfmito/R4 | R | GGATCTCCTGCAAATGTTGG |  |
| Pfmito/F5 | F | CAACATTTGCAGGAGATCCA | 833bp |
| Pfmito/R5 | R | TGTTCAATGGACATGGGTAA |  |
| Pfmito/F6 | F | AATAAATTACCCATGTCCATTGAA | 915bp |
| Pfmito/R6 | R | GGAACAGAATAATCTCTAGCACCA |  |
| Pfmito/F7 | F | TTAGCAGAACAAAGAAGTTTAACAAC | 833bp |
| Pfmito/R7 | R | GGTTATAGCCATGTCTCCATGA |  |
|  |  |  |  |
| **Cox I gene (nested PCR):** |  |  |  |
| PfCox1/F1 (1st round) | F | GCCCTATTACCATACAAGAGATCG | 1000bp |
| PfCox1/R1 (1st round) | R | TGACTAATTACTCCAAAAGCAGGT |  |
| PfCox1/F2 (2nd round) | F | TCTGGTATTTTGGACATCCTGA | 794bp |
| PfCox1/R2 (2nd round) | R | TGTTCAATGGACATGGGTAA |  |
|  |  |  |  |
| **Cox III gene:** |  |  |  |
| PfCox3/F | F | TGTCTATTCGTACAATTATTCAT | 997bp |
| PfCox3/R | R | CGTGACGAGCGGTGTGTA |  |
|  |  |  |  |
| **Cyt b gene (nested PCR):** |  |  |  |
| DW2 (1st round) | F | TAATGCCTAGACGTATTCCTGATTATCCAG | 1253bp |
| DW4 (1st round) | R | TGTTTGCTTGGGAGCTGTAATCATAATGTG |  |
| CYTb1 (2nd round) | F | CTCTATTAATTTAGTTAAAGCACA | 939pb |
| CYTb2 (2nd round) | R | ACAGAATAATCTCTAGCACC |  |
