# Supplementary material for: A New Malaria Agent in African Hominids
Source: PLoS Pathog. 2009 May 29;5(5):e1000446. doi: 10.1371/journal.ppat.1000446 (PMC2680981; doi:10.1371/journal.ppat.1000446)
Supplement: Text S1 — Supporting figures and tables. (1.05 MB DOC) [file ppat.1000446.s006.doc]

# Supporting Information Files

**A New Malaria Agent in African Hominids**

Benjamin Ollomo, Patrick Durand, Franck Prugnolle†, Emmanuel Douzery, Céline Arnathau, Dieudonné Nkoghe, Eric Leroy, François Renaud†

- †**To whom correspondence should be addressed. E-mail:** [prugnoll@mpl.ird.fr](mailto:prugnoll@mpl.ird.fr); [frrenaud@mpl.ird.fr](mailto:frrenaud@mpl.ird.fr)

**This file includes**

**Figs. S1 to S3**

**Tables S1 to S2**

**Supporting Figures**

**
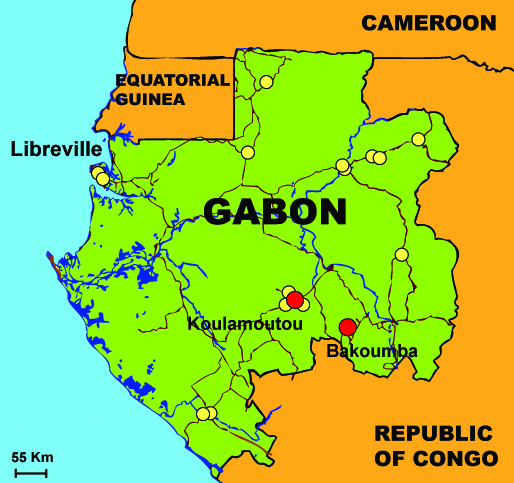
**

**Figure S1. Location of the 17 sampled chimpanzees (*Pan troglodytes*) in Gabon**. Each circle represents a unique sample. The fifteen uninfected chimpanzees are shown in yellow and the two infected ones in red. These latter two were collected in the villages of Koulamoutou (Ogooué-Lolo province) and Bakoumba (Haut Ogooué province), respectively.

10 20 30 40 50 60 70 80 90 100

....|....|....|....|....|....|....|....|....|....|....|....|....|....|....|....|....|....|....|....|

*P.falciparum* AAGCTTTTGGTATCTCGTAATGTAGAACAATATTGAGTTGACCGTCAAATCCTTTTCATTAAAAGAGTGGATTAAATGCCCAGCCAACACCATCCAATTT

*P.reichenowi* .........................................................................T..........................

*P. sp_K*  ......................................................................------------------------------

110 120 130 140 150 160 170 180 190 200

....|....|....|....|....|....|....|....|....|....|....|....|....|....|....|....|....|....|....|....|

*P.falciparum* GATTGGGAATTATCTGTGTTACAAATTTTTGATCCCAGGCTGGTAAAAAATGTAAACTTTTAGCCCATAAGAATAGAAACAGATGCCAGGCCAATAACTC

*P.reichenowi* ..............................................C.....................................................

*P. sp_K*  ----------------------------------------......TT..................G.................................

210 220 230 240 250 260 270 280 290 300

....|....|....|....|....|....|....|....|....|....|....|....|....|....|....|....|....|....|....|....|

*P.falciparum* AAACAGAGCTATGACGCTATCAATTTTTAGCAAGACGGATAAATTTTTCATAGAACTTAACGTATCATCATCCATGCAAAGATAAAACGGTAGATAGGGA

*P.reichenowi* ....................................................................................................

*P. sp_K*  ...T......................A.-..........C...A...............GT.......................................

310 320 330 340 350 360 370 380 390 400

....|....|....|....|....|....|....|....|....|....|....|....|....|....|....|....|....|....|....|....|

*P.falciparum* ACAAACTGCCTCAAGACGTTCTTAACCCAGCTCACGCATCGCTTCTAACGGTGAACTCTCATTCCAATGGAACCTTGTTCAAGTTCAAATAGATTGGTAA

*P.reichenowi* ....................................................................................................

*P. sp_K*  ....................................................................................................

410 420 430 440 450 460 470 480 490 500

....|....|....|....|....|....|....|....|....|....|....|....|....|....|....|....|....|....|....|....|

*P.falciparum* GGTATAGTGTTTACTATCAAATGAAACAATGTGTTCCACCGCTAGTGTTTGCTTCTAACATTCCACTTGCTTATAACTGTATGGACGTAACCTCCAGGCA

*P.reichenowi* ...G................................................................................................

*P. sp_K*  ............................T.......................................................................

510 520 530 540 550 560 570 580 590 600

....|....|....|....|....|....|....|....|....|....|....|....|....|....|....|....|....|....|....|....|

*P.falciparum* AAGAAAATGACCGGTCAAAACGGAATCAATTAACTATGGATAGCTGATACTATCAATTTATCATTACTCAAGTCAGCATAGTATATATGAAGGTTTCTAT

*P.reichenowi* ....................................................................................................

*P. sp_K*  ................................................TM...............................K.W................

610 620 630 640 650 660 670 680 690 700

....|....|....|....|....|....|....|....|....|....|....|....|....|....|....|....|....|....|....|....|

*P.falciparum* GGAAACACACTTCCCTTCTCGCCATTTGATAGCGGTTAACCTTTCCTTTTCCTTACGTACTCTAGCTATGAACACAATTGTCTATTCGTACAATTATTCA

*P.reichenowi* .................................................................................A..A...............

*P. sp_K*  .............................................................................C...A..................

710 720 730 740 750 760 770 780 790 800

....|....|....|....|....|....|....|....|....|....|....|....|....|....|....|....|....|....|....|....|

*P.falciparum* TATATATAT--TTGAAACAGGACATACATGTTCATTTATTCTGAATAGAATAAGAACTCTATAAATAACCAGACTATTTCAACAAAATGCCAATATAAAA

*P.reichenowi* .........--...............TC.....G..................................................................

*P. sp_K*  .........AT...............T..............A...................C.........T.TA.C.....................T.

810 820 830 840 850 860 870 880 890 900

....|....|....|....|....|....|....|....|....|....|....|....|....|....|....|....|....|....|....|....|

*P.falciparum* TTGTAATTTGATCAGTGTGAGGTATAACAATATATGATATACCGAAAGAATTTATAAACCATTCGGTAGAAGTATCATATATTTCTATTATTCTTATAAA

*P.reichenowi* ................A...................................ACA.............TT.A.........C...G..............

*P. sp_K*  ................A..T....C..TT..........CT..A...A..C.A.A.GC......T...TTT..G.......C.....CC...........

910 920 930 940 950 960 970 980 990 1000

....|....|....|....|....|....|....|....|....|....|....|....|....|....|....|....|....|....|....|....|

*P.falciparum* GTATATTATTAATAATAATAAACCTATTACTACATGAGAAAAATGTAATCCTGTAACACAATAAAATAATGTAGTATATACAGTATCATTTATATGATAT

*P.reichenowi* ........A.................C..T...............C.................G..................T.............G...

*P. sp_K*  ...A.......................A.T.....................A..T..................C......TT........A....A....

1010 1020 1030 1040 1050 1060 1070 1080 1090 1100

....|....|....|....|....|....|....|....|....|....|....|....|....|....|....|....|....|....|....|....|

*P.falciparum* GATAAATGTAAATACTCTGTAGTTTGTAGAGATGCAAAACATTCTCCTAATAAGTATATTATACAAATAATACTAGAGATTTCAAAACTCATTCCTTTTT

*P.reichenowi* .....G......................A......................................C.........A......................

*P. sp_K*  ..A.G.........T..A.C......C.AT.............GA.....A.TA..C.A....A.T........G..T.....T........A.T...C.

1110 1120 1130 1140 1150 1160 1170 1180 1190 1200

....|....|....|....|....|....|....|....|....|....|....|....|....|....|....|....|....|....|....|....|

*P.falciparum* CTATAAATACTTGTAAACATGCAGTCATACATGATGCACTAGCTAATATAAATGTAATTGTTAAGATTAACATTCTTGATGAAGTAATGATAATACCTTC

*P.reichenowi* ...A............G....................................................................G..A.C.........

*P. sp_K*  .........A......G.....T..T....................A........T.......GT..A.G............G.....T..T........

1210 1220 1230 1240 1250 1260 1270 1280 1290 1300

....|....|....|....|....|....|....|....|....|....|....|....|....|....|....|....|....|....|....|....|

*P.falciparum* ATTACTTAATGGATATGGTGATAAACTAAAATGTAATATACCCCAAAAATATGTAAAGAATAATAAAGCTTCTGATATTATGATAGATAACATACCAGAA

*P.reichenowi* .....A......G..........G.........C.........................................G.....A..............T...

*P. sp_K*  .C...C...A.......................A.GA........G..........TA....................A..A........T........T

1310 1320 1330 1340 1350 1360 1370 1380 1390 1400

....|....|....|....|....|....|....|....|....|....|....|....|....|....|....|....|....|....|....|....|

*P.falciparum* GTTAAAGATGAAAATACAGAATAAAAACTTTCTCGAATAGAATATACAAATATTAATAGGATTATAGGGTTAAATGTAAATAATATCCCTACAGAAAAGT

*P.reichenowi* ............................................A.............A...........................A...........A.

*P. sp_K*  ........G......GT......T..........T...T.....A........G.TG.AA....A...A.................A.....T.....A.

1410 1420 1430 1440 1450 1460 1470 1480 1490 1500

....|....|....|....|....|....|....|....|....|....|....|....|....|....|....|....|....|....|....|....|

*P.falciparum* ATTTTAAAGATGTACCATATAATGATGTTAATGCAGGATATGAAACTAGATGTGCTTTTATATTTGATAAATTACTAAATAAAATAAAT----------T

*P.reichenowi* ........................................G.....A.T........................................----------.

*P. sp_K*  ......G..T....................TG.....G.....T....T....................G...................AACATGTTAT.

1510 1520 1530 1540 1550 1560 1570 1580 1590 1600

....|....|....|....|....|....|....|....|....|....|....|....|....|....|....|....|....|....|....|....|

*P.falciparum* TATAAGAACGGTGAGATAATGTGCCGTAAACATATAACGGTAAGAAGGTTCGCCGGGGATAACAGGTTATAGTATATATAGAGCTCTAATCTTTATATAC

*P.reichenowi* ................G.G.................................................................................

*P. sp_K*  ...............GG.G.............................................................................----

1610 1620 1630 1640 1650 1660 1670 1680 1690 1700

....|....|....|....|....|....|....|....|....|....|....|....|....|....|....|....|....|....|....|....|

*P.falciparum* TATTGGCACCTCCATGTCGTCTCATCGCAGCCTTGCAATAAATAATATCTAGCGTGTATTGTTGCCTTGTACACACCGCTCGTCACGCAATATCAATATA

*P.reichenowi* ..........................................G.....T............................................TTT....

*P. sp_K*  ----------------------------------------------------------------------------------------------------

1710 1720 1730 1740 1750 1760 1770 1780 1790 1800

....|....|....|....|....|....|....|....|....|....|....|....|....|....|....|....|....|....|....|....|

*P.falciparum* CTGGGTATAGAACTCCAGGCGTTAACCTGTAGAGTTGAGATGGAAACAGCCGGAAAGGTAATTTTACGCCCTTAACGTAAAGATCATTTATGAAATAGAT

*P.reichenowi* T..A.A..............................................................................T...............

*P. sp_K*  ----------------------------------------------------------------------------------------------------

1810 1820 1830 1840 1850 1860 1870 1880 1890 1900

....|....|....|....|....|....|....|....|....|....|....|....|....|....|....|....|....|....|....|....|

*P.falciparum* TAGCATGGGACTAAAAAATGTTATGTTGTTGGTTTAAGCCCTATTACCATACAAGAGATCGCGTACTTTGGACCGAATAAAGCTGTGAGGAAACTACATT

*P.reichenowi* ....................................................................................................

*P. sp_K*  -------------------------------------------------------------------..........AT.....................

1910 1920 1930 1940 1950 1960 1970 1980 1990 2000

....|....|....|....|....|....|....|....|....|....|....|....|....|....|....|....|....|....|....|....|

*P.falciparum* AAAGGAACTCGACTGGCCTACACTATAAGAACGAACGCTTTTAACGCCTGACATGGATGGATAATACTCGACTCTTCCAAAGTATAACCGCTGTCGCTGG

*P.reichenowi* ....................................................................................................

*P. sp_K*  ......................................................................G.............................

2010 2020 2030 2040 2050 2060 2070 2080 2090 2100

....|....|....|....|....|....|....|....|....|....|....|....|....|....|....|....|....|....|....|....|

*P.falciparum* GACTGTATGGATCAAATATTTCTCATTTATATCCGAGCCTCATGTTATTTTTATTGTTTTAAATAGATATTCACTTATTACAAATTGTAACCATAAAACT

*P.reichenowi* ....................................................................................................

*P. sp_K*  ......................CA..................GA....G...........T.........A.............................

2110 2120 2130 2140 2150 2160 2170 2180 2190 2200

....|....|....|....|....|....|....|....|....|....|....|....|....|....|....|....|....|....|....|....|

*P.falciparum* TTAGGATTATACTATTTATGGTTTTCATTTTTATTTGGTAGTTATGGATTTTTATTATCAGTAATACTACGTACTGAATTATATTCTTCATCTTTAAGAA

*P.reichenowi* .............................C...........C................................A........................G

*P. sp_K*  .............................C.............................T......T.......A..G..............A.......

2210 2220 2230 2240 2250 2260 2270 2280 2290 2300

....|....|....|....|....|....|....|....|....|....|....|....|....|....|....|....|....|....|....|....|

*P.falciparum* TAATTGCACAAGAAAATGTAAATCTATATAATATGATATTTACAATTCACGGAATAATTATGATTTTTTTCAATATAATGCCAGGATTATTCGGAGGATT

*P.reichenowi* ....A..T......................................A...........A.........................................

*P. sp_K*  .T...............C............................A....................C..............T........T........

2310 2320 2330 2340 2350 2360 2370 2380 2390 2400

....|....|....|....|....|....|....|....|....|....|....|....|....|....|....|....|....|....|....|....|

*P.falciparum* TGGTAATTACTTTCTACCTATTTTATGTGGATCTCCAGAATTAGCATATCCTAGAATTAATAGTATATCTTTACTGTTACAACCAATTGCTTTTGTTTTA

*P.reichenowi* .............T....C.................................................................................

*P. sp_K*  .............A........C.............T..G..............................C..T.A........T...........A...

2410 2420 2430 2440 2450 2460 2470 2480 2490 2500

....|....|....|....|....|....|....|....|....|....|....|....|....|....|....|....|....|....|....|....|

*P.falciparum* GTTATATTATCTACTGCAGCAGAATTTGGTGGTGGAACTGGATGGACTTTATATCCACCATTAAGTACATCTTTAATGTCATTATCTCCTGTAGCTGTAG

*P.reichenowi* ....................................................................................................

*P. sp_K*  ..G..CC..........T..T..............T...................................AC................G..........

2510 2520 2530 2540 2550 2560 2570 2580 2590 2600

....|....|....|....|....|....|....|....|....|....|....|....|....|....|....|....|....|....|....|....|

*P.falciparum* ATGTAATAATTTTTGGTTTATTAGTATCTGGAGTCGCTAGTATTATGTCTTCATTAAATTTTATTACTACAGTAATGCATTTAAGAGCAAAAGGATTAAC

*P.reichenowi* ............................A.....A..................................................G........T.....

*P. sp_K*  .......T..A..A...........T......A.A..A..............T.................T.................T.....T.....

2610 2620 2630 2640 2650 2660 2670 2680 2690 2700

....|....|....|....|....|....|....|....|....|....|....|....|....|....|....|....|....|....|....|....|

*P.falciparum* ACTTGGTATATTAAGTGTTTCTACATGGTCATTGATCATTACATCAGGAATGTTATTGCTAACACTACCGGTTTTAACTGGAGGAGTATTAATGTTATTA

*P.reichenowi* .....................A...........A..............T......C.A......T....A..............................

*P. sp_K*  ...C........G........A.........A.AT.A....................AT..........T..CC....A.....Y...............

2710 2720 2730 2740 2750 2760 2770 2780 2790 2800

....|....|....|....|....|....|....|....|....|....|....|....|....|....|....|....|....|....|....|....|

*P.falciparum* TCAGACTTACATTTTAATACTTTATTTTTTGACCCAACATTTGCAGGAGATCCAATATTATATCAACATTTATTCTGGTTTTTTGGACATCCTGAAGTAT

*P.reichenowi* ...........C..........................................................................T.............

*P. sp_K*  ..T..........................C..T........---------------------------------.....A....................

2810 2820 2830 2840 2850 2860 2870 2880 2890 2900

....|....|....|....|....|....|....|....|....|....|....|....|....|....|....|....|....|....|....|....|

*P.falciparum* ACATTTTAATATTACCTGCTTTTGGAGTAATTAGTCATGTAATTTCTACTAATTATTGCAGAAATCTATTTGGTAATCAATCTATGATACTTGCTATGGG

*P.reichenowi* .T.....................................................C..T.........................................

*P. sp_K*  .T..............A..A.....C..TG....C..C.....C....AC........T....G.T..................................

2910 2920 2930 2940 2950 2960 2970 2980 2990 3000

....|....|....|....|....|....|....|....|....|....|....|....|....|....|....|....|....|....|....|....|

*P.falciparum* ATGTATAGCTGTTTTAGGAAGCTTAGTATGGGTACATCATATGTACACTACTGGTTTAGAAGTTGATACTAGAGCTTATTTTACTTCGACTACCATTTTA

*P.reichenowi* ...................................................A...................................T........A...

*P. sp_K*  T............C.......T.......................T.....A...C.............A........C.....A..T.....T......

3010 3020 3030 3040 3050 3060 3070 3080 3090 3100

....|....|....|....|....|....|....|....|....|....|....|....|....|....|....|....|....|....|....|....|

*P.falciparum* ATATCAATACCTACCGGTACAAAAGTATTTAACTGGATATGTACATATATGAGTAGTAATTTTGGTATGATACACAGCTCTTCATTATTGTCATTATTAT

*P.reichenowi* ..............T...........................................................T........C................

*P. sp_K*  ..T..T..T.....A.....T.....G.....T...................................A..T..T..T......C....A..........

3110 3120 3130 3140 3150 3160 3170 3180 3190 3200

....|....|....|....|....|....|....|....|....|....|....|....|....|....|....|....|....|....|....|....|

*P.falciparum* TTATATGTACATTTACATTTGGAGGTACTACTGGAGTTATATTAGGTAATGCTGCCATTGATGTAGCATTACATGACACATATTATGTTATTGCTCATTT

*P.reichenowi* ......................G...........G.....C..............T............................................

*P. sp_K*  ................T.....T.....A..A..T..A...C..........A..T..C...A.............T...........A..C.....C..

3210 3220 3230 3240 3250 3260 3270 3280 3290 3300

....|....|....|....|....|....|....|....|....|....|....|....|....|....|....|....|....|....|....|....|

*P.falciparum* CCATTTTGTACTATCAATTGGTGCAATTATTGGATTATTTACAACTGTAAGTGCATTTCAAGATAATTTCTTTGGTAAAAACTTACGTGAAAATTCTATT

*P.reichenowi* ..................C...........C....................................C.............T.....A...........C

*P. sp_K*  T.........T....C...........A.....................................................T.....A.....C..AG.A

3310 3320 3330 3340 3350 3360 3370 3380 3390 3400

....|....|....|....|....|....|....|....|....|....|....|....|....|....|....|....|....|....|....|....|

*P.falciparum* GTAATACTATGGTCAATGTTATTTTTTGTAGGTGTAATATTAACATTTTTACCTATGCATTTTTTAGGATTTAATGTAATGCCTAGACGTATTCCTGATT

*P.reichenowi* A.................C.................................................................................

*P. sp_K*  A..G..T........T..C.......C..T..G.....TC....C..............C..C.....................................

3410 3420 3430 3440 3450 3460 3470 3480 3490 3500

....|....|....|....|....|....|....|....|....|....|....|....|....|....|....|....|....|....|....|....|

*P.falciparum* ATCCAGACGCTTTAAATGGATGGAATATGATTTGTTCTATTGGGTCAACAATGACTTTATTTGGTTTACTAATTTTTAAATAATATTACTATTTATTGTT

*P.reichenowi* ....................................................................................................

*P. sp_K*  ...........................................A.Y......................T................C..............

3510 3520 3530 3540 3550 3560 3570 3580 3590 3600

....|....|....|....|....|....|....|....|....|....|....|....|....|....|....|....|....|....|....|....|

*P.falciparum* TTTATGAACTTTTACTCTATTAATTTAGTTAAAGCACACTTAATAAATTACCCATGTCCATTGAACATAAACTTTTTATGGAATTACGGATTCCTTTTAG

*P.reichenowi* ..A...................................................................................T........A....

*P. sp_K*  .C..........C.........................T.....T.....................------------........T.............

3610 3620 3630 3640 3650 3660 3670 3680 3690 3700

....|....|....|....|....|....|....|....|....|....|....|....|....|....|....|....|....|....|....|....|

*P.falciparum* GAATAATATTTTTTATTCAAATTATAACAGGTGTATTTTTAGCAAGTCGATATACACCAGATGTTTCATATGCATATTATAGTATACAACACATTTTAAG

*P.reichenowi* ....C.......................T...................................A...................................

*P. sp_K*  ....T.........G.......A..T..T....................T....................C.....C.......................

3710 3720 3730 3740 3750 3760 3770 3780 3790 3800

....|....|....|....|....|....|....|....|....|....|....|....|....|....|....|....|....|....|....|....|

*P.falciparum* AGAATTATGGAGTGGATGGTGTTTTAGATACATGCACGCAACAGGTGCTTCTCTTGTATTTTTATTAACATATCTTCATATTTTAAGAGGATTAAATTAC

*P.reichenowi* ..............................T.....T....................................T.G.......................T

*P. sp_K*  ...GC..............GC.........T.....T...........A........................T.A........................

3810 3820 3830 3840 3850 3860 3870 3880 3890 3900

....|....|....|....|....|....|....|....|....|....|....|....|....|....|....|....|....|....|....|....|

*P.falciparum* TCATATATGTATTTACCATTATCATGGATATCTGGATTGATTTTATTTATGATATTTATTGTAACTGCTTTCGTTGGTTATGTCTTACCATGGGGTCAAA

*P.reichenowi* ................................C.......................C.....T....................T................

*P. sp_K*  .....C.....C....................A.....A..........................A.....T........C..AC..........A....

3910 3920 3930 3940 3950 3960 3970 3980 3990 4000

....|....|....|....|....|....|....|....|....|....|....|....|....|....|....|....|....|....|....|....|

*P.falciparum* TGAGTTATTGGGGTGCAACTGTAATTACTAACTTGTTATCCTCTATTCCAGTAGCAGTAATTTGGATATGTGGAGGATATACTGTGAGTGATCCTACAAT

*P.reichenowi* ...................A..............A.......................T.........................................

*P. sp_K*  ....C..............A...........T........T..C...........T..T.................T.....A..C..............

4010 4020 4030 4040 4050 4060 4070 4080 4090 4100

....|....|....|....|....|....|....|....|....|....|....|....|....|....|....|....|....|....|....|....|

*P.falciparum* AAAACGATTTTTTGTACTACATTTTATCTTACCATTTATTGGATTATGTATTGTATTTATACATATATTTTTCTTACATTTACATGGTAGCACAAATCCT

*P.reichenowi* ...............TT..........A........................................................................

*P. sp_K*  ............C..TT....C.....T.......................C...........................C.............T.....A

4110 4120 4130 4140 4150 4160 4170 4180 4190 4200

....|....|....|....|....|....|....|....|....|....|....|....|....|....|....|....|....|....|....|....|

*P.falciparum* TTAGGGTATGATACAGCATTAAAAATACCCTTTTATCCAAATCTATTAAGTCTTGATGTTAAAGGATTTAATAATGTTATAATTTTATTTCTAATACAAA

*P.reichenowi* .............................................................................A..C...................

*P. sp_K*  ................................C..........................................A....T........CT....T....

4210 4220 4230 4240 4250 4260 4270 4280 4290 4300

....|....|....|....|....|....|....|....|....|....|....|....|....|....|....|....|....|....|....|....|

*P.falciparum* GTTTATTTGGAATTATACCTTTATCACATCCTGATAATGCTATCGTAGTAAATACATATGTTACTCCATCTCAAATTGTACCTGAATGGTACTTTCTACC

*P.reichenowi* ...........................................T................................A.......................

*P. sp_K*  .............A.................A...........TA.............C........G...........T...........T...T....

4310 4320 4330 4340 4350 4360 4370 4380 4390 4400

....|....|....|....|....|....|....|....|....|....|....|....|....|....|....|....|....|....|....|....|

*P.falciparum* ATTTTATGCAATGTTAAAAACTGTTCCAAGTAAACCAGCTGGTTTAGTAATTGTATTATTATCATTACAATTATTATTCTTATTAGCAGAACAAAGAAGT

*P.reichenowi* .......................................................................................C............

*P. sp_K*  .....................A.....T........G.....G.....T........................C.............T............

4410 4420 4430 4440 4450 4460 4470 4480 4490 4500

....|....|....|....|....|....|....|....|....|....|....|....|....|....|....|....|....|....|....|....|

*P.falciparum* TTAACAACTATAATTCAATTTAAAATGATTTTTGGTGCTAGAGATTATTCTGTTCCTATTATATGGTTTATGTGTGCATTCTATGCTTTATTATGGATTG

*P.reichenowi* ....................................................................................................

*P. sp_K*  ..............A.................C...................................................................

4510 4520 4530 4540 4550 4560 4570 4580 4590 4600

....|....|....|....|....|....|....|....|....|....|....|....|....|....|....|....|....|....|....|....|

*P.falciparum* GATGTCAATTACCACAAGATATATTCATTTTATATGGTCGATTATTTATTGTATTATTTTTCTGTAGTGGTTTATTTGTACTTGTTCATTATAGACGAAC

*P.reichenowi* ......................T......................................................................A......

*P. sp_K*  .............C.....C..T.......................................A......................C.......A.A....

4610 4620 4630 4640 4650 4660 4670 4680 4690 4700

....|....|....|....|....|....|....|....|....|....|....|....|....|....|....|....|....|....|....|....|

*P.falciparum* ACATTATGATTACAGCTCCCAAGCAAACATATAATATTACAAGATTGTGATAAGATGACATTTCTGAGTATTGAGCGGAACAAATCAGACCGTAAGGTTA

*P.reichenowi* ....................................................................................G...............

*P. sp_K*  ..........................................................................A.........A...............

4710 4720 4730 4740 4750 4760 4770 4780 4790 4800

....|....|....|....|....|....|....|....|....|....|....|....|....|....|....|....|....|....|....|....|

*P.falciparum* TAATTATGTACTATGATTGGAAAATATAACTATAGTTACCATAGCTGTAGATGGATGCTTCGATATATAGTATATTACAGTATCAATCGGATTTACATGC

*P.reichenowi* .............................T......................................................................

*P. sp_K*  ........C.T......A.......G...A...................................ATA.A...................T..........

4810 4820 4830 4840 4850 4860 4870 4880 4890 4900

....|....|....|....|....|....|....|....|....|....|....|....|....|....|....|....|....|....|....|....|

*P.falciparum* TCAGCCGCCAAAAACTATAACGATATTATTACCGTACAAGCCGTTAGCAAGACATGATAGGGAGTTGGCAAGTTAAAGAAGTTCTGGTTTATAATAGATA

*P.reichenowi* ....................................................................................................

*P. sp_K*  ..............-.....................................................................................

4910 4920 4930 4940 4950 4960 4970 4980 4990 5000

....|....|....|....|....|....|....|....|....|....|....|....|....|....|....|....|....|....|....|....|

*P.falciparum* CGTTATTAATGTTAGGATGTATGGGATATTTGTAGTACACCTTGATTGGTTTTACTATTTATATTTATCGATAAATGTTCGGTATTGCATGCCTGGTGTT

*P.reichenowi* ............................................................T.......................................

*P. sp_K*  ....TA......................C...........................T.......A.T.....G.G.........................

5010 5020 5030 5040 5050 5060 5070 5080 5090 5100

....|....|....|....|....|....|....|....|....|....|....|....|....|....|....|....|....|....|....|....|

*P.falciparum* TTTAATATAGACGCTGACTTCCTGGCTAAACTTCCCAATGATATATCTTCCAAATAGATTTCGCAGAAAACCGTCTATATTCATGTTTGATTGACCTTTA

*P.reichenowi* ....................................................................................................

*P. sp_K*  .....................................T..............................................................

5110 5120 5130 5140 5150 5160 5170 5180 5190 5200

....|....|....|....|....|....|....|....|....|....|....|....|....|....|....|....|....|....|....|....|

*P.falciparum* ACCACTAATTACGAATCTTCCAAGAATATTTTAAGAGTCCAAGGTTCGGTCTATTATTTTCCTGTTCTGTAATTAGATCACATGTTTTATAGTTCATGGA

*P.reichenowi* ......................................................C.......G....G.....A..........................

*P. sp_K*  ..................................................Y...ATA...........................................

5210 5220 5230 5240 5250 5260 5270 5280 5290 5300

....|....|....|....|....|....|....|....|....|....|....|....|....|....|....|....|....|....|....|....|

*P.falciparum* GACATGGCTATAACCACTATTCATAGAGACAACTAATGGAATCTCTCTCGATTTCCAGATGTTGAGTTACTAAGAGGATTCTCTCCACACTTCAATTCGT

*P.reichenowi* ....................................................................................................

*P. sp_K*  .....C.------------------------------------------..A................................................

5310 5320 5330 5340 5350 5360 5370 5380 5390 5400

....|....|....|....|....|....|....|....|....|....|....|....|....|....|....|....|....|....|....|....|

*P.falciparum* ACTTCCACTACCAGAATATACTCTCCTGTTCTAAAATTCTAGGATTTTTCGCGTTTTTTCAGGAGAAATCCGTATATCGATGTCTTTTAATCAATGCTAT

*P.reichenowi* .......................................................................................A............

*P. sp_K*  ............................................C.............-............................A..A.........

5410 5420 5430 5440 5450 5460 5470 5480 5490 5500

....|....|....|....|....|....|....|....|....|....|....|....|....|....|....|....|....|....|....|....|

*P.falciparum* TGGATTCAACGTCCAGGACTTCCTGACGCTTAATAACGATTTCTACTTCCAGCAGCCATTTTTGGTTCAGCTACAAGTTCACTGTCAACTACCATGTTAC

*P.reichenowi* .............................................................A-.....................................

*P. sp_K*  ................................G............................A-.....................................

5510 5520 5530 5540 5550 5560 5570 5580 5590 5600

....|....|....|....|....|....|....|....|....|....|....|....|....|....|....|....|....|....|....|....|

*P.falciparum* GACTTCGCACCGACTGTTTCTTTTACCTCACGAGTCGATCAGGAAGGTTTCATCCTTAAATCTCGTAACCATGCCAACACATAAGAACTTTTAGGGAAGT

*P.reichenowi* ....................................................................................................

*P. sp_K*  ....................................................................................................

5610 5620 5630 5640 5650 5660 5670 5680 5690 5700

....|....|....|....|....|....|....|....|....|....|....|....|....|....|....|....|....|....|....|....|

*P.falciparum* TAAGGTGCTCAGGGTCTTACCGTCGGGCCGTATGATTCCACATATTCATGGATAATTCTATTTATTAGGAGTCTCACACTAGCGACAATGGGGAAGTCGT

*P.reichenowi* ....................................................................................................

*P. sp_K*  ....................................................................................................

5710 5720 5730 5740 5750 5760 5770 5780 5790 5800

....|....|....|....|....|....|....|....|....|....|....|....|....|....|....|....|....|....|....|....|

*P.falciparum* TACACCGTTCATGCAGGACGGAGATTACCCGACAAGGAATTTTGCTACCTTAGGACCGTTTAAAATACAGCCGCCGTTTATCATTGATGCCGGGCAGATG

*P.reichenowi* ....................................................................................................

*P. sp_K*  ..............................................................T...................G.A...............

5810 5820 5830 5840 5850 5860 5870 5880 5890 5900

....|....|....|....|....|....|....|....|....|....|....|....|....|....|....|....|....|....|....|....|

*P.falciparum* TCAGTAACTTGAAATATTCATCAGAATTATCAGTGACTTGTGTTGTAACCTTACAGACGCTTCCAGTAATTTAACTTCTTATAAATGGAAGCGCCGGTTT

*P.reichenowi* ......................................................................C.............................

*P. sp_K*  ....................................................................................................

5910 5920 5930 5940 5950 5960 5970

....|....|....|....|....|....|....|....|....|....|....|....|....|....|....|....

*P.falciparum* CCCGGGTATCCAATCCAGTGCTCCATTCAAGGCATAGAGACTCAGCCTATGTTCAACTTTG------------------

*P.reichenowi* .............................................................TAGAGTTATATTATAATA

*P. sp_K*  .............................................................------------------

**Figure S2.** **Multiple sequence alignment of the whole mitochondrial DNA of the three species *P. falciparum (3D7 strain)*, *P. reichenowi* and *P. sp_*K using CLUSTAL W (v. 1.81)**. A dot indicates an identical nucleotide and a dash indicates a gap compared to the *P. falciparum* reference sequence. Degenerate nucleotides as follows: W = AT, Y = CT, K = GT, M = AC.

**100**

**100**

**88**

**
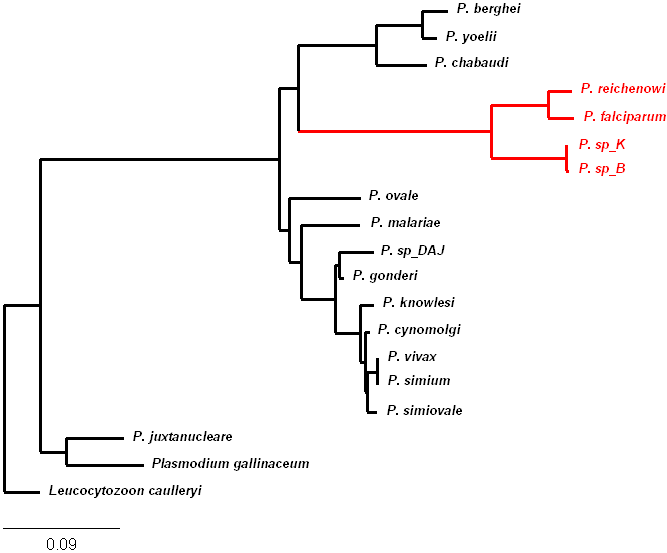
**

**Figure S3. Phylogenetic relationships among *Plasmodium* species (including *P. sp_K and P. sp_B*)**. The phylogram presented here was reconstructed by a Maximum Likelihood approach from partial *Cyt b* DNA sequence data (866 nt). Bootstrap values obtained are only shown (in blue) for the nodes inside the African Great Apes - Human lineage (represented in red). *Leucocytozoon caulleryi* was used as outgroup. Scale bar shows 0.09 substitutions per site.

**Supporting tables**

**table S1. Parasite species used in this study with GenBank accession numbers and a description of their natural hosts.**

| **Species** | **Natural host** | **GenBank Accession numbers** |
| --- | --- | --- |
| *Plasmodium falciparum (3D7)* | Human | AY282930 |
| *P. vivax* | Human | NC_007243 |
| *P. ovale* | Human | AB354571 |
| *P. malariae* | Human | AB354570 |
| *P. reichenowi* | Chimpanzee and Gorilla | NC_002235 |
| *P. sp_K* | Chimpanzee | This study |
| *P. sp_B* | Chimpanzee | This study |
| *P. gonderi* | African Old World Monkey | AB434918 |
| *P. sp. DAJ-2004* | African Old World Monkey | AY800112 |
| *P. knowlesi* | Asian Old World Monkey and Human | AY722797 |
| *P. simium* | New World Monkey | NC_007233 |
| *P. cynomolgi* | Asian Old World Monkey | AY800108 |
| *P. simiovale* | Asian Old World Monkey | AB434920 |
| *P. chabaudi* | Rodent | AF014116 |
| *P. yoelii* | Rodent | M29000 |
| *P. berghei* | Rodent | AF014115 |
| *P. juxtanucleare* | Bird | AB250415 |
| *P. gallinaceum* | Bird | AB250690 |
| *Leucocytozoon caulleryi* | Bird | AB302215 |

**table S2.** **Amplification primers of the mitochondrial genome**. Primers used in this study for the amplification of the whole mitochondrial genome and the three genes (*Cox* I, III and *Cyt* b) separately for *P.sp_B and P.sp_K* based on the mitochondrial genome sequence of *P. falciparum* 3D7 strain (GenBank Acc. no. AY282930)*.* The fragment sizes are also estimated from the *P. falciparum* 3D7 sequence. mt: mitochondrial; F: Forward; R: Reverse; bp: base pairs; *Cox* I and *Cox* III: cytochrome oxydase I and III, respectively; *Cyt* b: cytochrome b. *Cox* I and *Cyt* b are amplified by nested PCR.

Primer Name Sense Sequence (5’🡪3’) Fragment size

Whole mt genome:

Pfmito/F1 F CTCTCTCGATTTCCAGATGTTG 821bp

Pfmito/R1 R GCTGGGCATTTAATCCACTC

Pfmito/F2 F ATTAAATGCCCAGCCAACAC 888bp

Pfmito/R2 R TGTGTTACAGGATTACATTTTTCTCA

Pfmito/F3 F AATACTCTGTAGTTTGTAGAGATG 885bp

Pfmito/R3 R CCTTTAATGTAGTTTCCTCA

Pfmito/F4 F TGGACCGAATAAAGCTGTGA 885bp

Pfmito/R4 R GGATCTCCTGCAAATGTTGG

Pfmito/F5 F CAACATTTGCAGGAGATCCA 833bp

Pfmito/R5 R TGTTCAATGGACATGGGTAA

Pfmito/F6 F AATAAATTACCCATGTCCATTGAA 915bp

Pfmito/R6 R GGAACAGAATAATCTCTAGCACCA

Pfmito/F7 F TTAGCAGAACAAAGAAGTTTAACAAC 833bp

Pfmito/R7 R GGTTATAGCCATGTCTCCATGA

*Cox* I gene (nested PCR):

PfCox1/F1 (1^st^ round) F GCCCTATTACCATACAAGAGATCG 1000bp

PfCox1/R1 (1^st^ round) R TGACTAATTACTCCAAAAGCAGGT

PfCox1/F2 (2^nd^ round) F TCTGGTATTTTGGACATCCTGA 794bp

PfCox1/R2 (2^nd^ round) R TGTTCAATGGACATGGGTAA

*Cox* III gene:

PfCox3/F F TGTCTATTCGTACAATTATTCAT 997bp

PfCox3/R R CGTGACGAGCGGTGTGTA

*Cyt* b gene (nested PCR):

DW2 (1^st^ round) F TAATGCCTAGACGTATTCCTGATTATCCAG 1253bp

DW4 (1^st^ round) R TGTTTGCTTGGGAGCTGTAATCATAATGTG

CYTb1 (2^nd^ round) F CTCTATTAATTTAGTTAAAGCACA 939pb

CYTb2 (2^nd^ round) R ACAGAATAATCTCTAGCACC
